# Associations between external learning contexts and college students’ learning resilience through academic self-efficacy and perceived campus belonging

**DOI:** 10.1038/s41598-026-51060-6

**Published:** 2026-05-02

**Authors:** Fangfang Wu, Huanyu Huang, Zirui Zhan

**Affiliations:** 1 College of Education, Zhejiang Normal University, Jinhua, 321004 Zhejiang China; 2 College of Physical Education and Health Science, Zhejiang Normal University, Jinhua, 321004 China; 3 Shanghai Academy of Fine Arts, Shanghai University, Shanghai, 200444 China

**Keywords:** Social cognitive theory, Triadic reciprocal determinism, Academic self-efficacy, Perceived campus belonging, Learning resilience, Education, Psychology, Psychology

## Abstract

In the digital age, university students’ sustained academic engagement and strong learning resilience in the face of increasing academic pressure and complex campus challenges are essential to the attainment of substantial academic achievement. At present, how to enhance students’ academic engagement and foster learning resilience has become a pressing issue for educational administrators. Although previous studies have examined multiple factors influencing academic engagement and resilience, they have largely emphasized the isolated effects of psychological traits on individual learning performance while overlooking the complex possibility that perceived external contexts, such as the learning environment, learning climate, and social relationships, may jointly shape learning resilience through psychological and emotional regulatory mechanisms. Therefore, this study focuses on the interaction among external contexts, internal affective drivers (academic self-efficacy and perceived campus belonging), and learning resilience. Using questionnaire survey data and structural equation modeling, this study examines the extent to which external contexts are associated with academic self-efficacy and perceived campus belonging, explores whether these internal affective drivers are statistically associated with learning resilience through mediating pathways, and constructs an “external context–affective drivers–learning resilience” model to identify potential explanatory pathways and provide evidence-based implications for educational management.

## Introduction

In the digital age, higher education has undergone a profound transformation. While digital platforms and ubiquitous internet access offer unprecedented learning resources, they have simultaneously plunged college students into a highly demanding learning environment characterized by cognitive overload and fragmented attention. Furthermore, the increasing reliance on digital interfaces has inadvertently weakened traditional, face-to-face interpersonal interactions, leading to a prevalent sense of "digital social isolation" among university students. Behavioral commitment, adaptability, and emotional regulation during the learning process directly influence academic engagement and the willingness to persist in learning^1–3^. However, under these dual cognitive and psychosocial pressures, students often find themselves navigating complex academic tasks in a state of emotional detachment, increasingly vulnerable to academic setbacks and self-doubt.

The consequences of this vulnerability are profound. When confronted with academic difficulties, intense competition, or prolonged negative emotions, students lacking adequate psychological buffers are highly susceptible to superficial learning, behavioral disengagement, or even severe academic burnout. Existing research indicates that academic engagement is closely associated with learning resilience^4^, and the two exhibit a bidirectional, mutually reinforcing relationship^5,6^. Therefore, rather than merely emphasizing surface-level academic engagement, it has become critically urgent for educational administrators to understand and cultivate students’ learning resilience. This refers to the psychological quality and capacity enabling individuals to rapidly recover, maintain goals, and proactively adjust strategies to continue engaging in learning when confronted with setbacks, difficulties, stress, and failure^7–9^. It provides the core emotional foundation that prevents students from abandoning their educational pursuits halfway.

However, learning resilience does not develop in isolation; it is shaped by multiple factors including the learning environment, climate, social networks, and individual characteristics^4,5,10^. According to social cognitive theory, when learning environment satisfy individuals’ fundamental psychological needs,such as autonomy, competence, and relatedness,they are more likely to develop intrinsic motivation, leading to higher levels of engagement and persistence^4,11^. Simultaneously, the learning climate and social relationships, as crucial components of the learning environment, play a central role in shaping students’ adaptive and regulable psychological states^12,13^. A positive learning climate and healthy peer-to-peer and teacher-student relationships not only enhance students’ interest in courses but also enable them to make positive subjective judgments regarding task completion^14,15^. In the context of the isolating digital age, we posit that supportive external contexts may shape students’ academic self-efficacy and perceived campus belonging, which in turn are associated with learning resilience. These internalized individual traits act as the crucial affective drivers that forge robust learning resilience.

To address this gap in the literature, this study focuses on the intricate generative mechanisms of learning resilience among college students. Using structural equation modeling (SEM), this research aims to: (1) examine the extent to which external contexts are associated with internal affective drivers (academic self-efficacy and perceived campus belonging); (2) explore whether these internal traits are linked to learning resilience through mediating pathways; and (3) construct and validate a comprehensive “External Context–Affective Drivers–Learning Resilience” model. By mapping these multidirectional pathways, this study seeks to provide evidence-based insights for educators and administrators regarding how campus support systems may be associated with students’ learning resilience in contemporary higher education settings.

## Literature review

### Social cognitive theory

The social cognitive theory, which was developed and improved by the American psychologist Albert Bandura in the 1970s, has become a broadly used psychological model in education to analyze the influences that can promote student learning behavior under complicated settings^7,16,17^. Social cognitive theory suggests a dynamic, interactive relationship between individual behavior, cognition and environment^8^. Human beings have a natural inclination to grow and explore actively. Individuals tend to feel more connected to their environment and have a high level of self-identity when external environments meet basic psychological requirements, which increases the likelihood of active participation in learning^10,18^. Nevertheless, the process of learning does not always go easy,people might come across obstacles and adversity, and sometimes, they may experience failure or exclusion. The strength of learning resilience of an individual is especially put to test when he or she has the courage to be able to regain confidence in learning after repeated setbacks.

The social cognitive theory states that the results are not affected or controlled by a sole variable. It confirms that there is a mutual interaction between individual characteristics, behavioral characteristics, and environmental characteristics^16,19^. As an example, a student who gets encouragement by teachers and acknowledgment by classmates throughout learning will have more learning reinforcement (strengthening learning motivation and learning behavior). On the contrary, if a student experiences repeated failures and long-term negative emotions associated with learning, they might start to experience self-doubt and self-abandonment thinking, making it hard to revive interest in learning. We also conduct our research on people ability to handle negative emotions, watching and investigating the exact ways to pinpoint leverage points of environment, individual characteristics, and behavior. Its purpose is to help education managers and students to regulate and intervene the learning in order to achieve positive learning outcomes. This theory highlights the importance of the idea that individuals can control their own behavior through self-control instead of being submissive to environmental factors^2,7^. Self-regulation allows individuals to keep up with the behaviors that are consistent with social norms or personal goals without any external rewards or punishment^9,20^. This implies that variables like learning environment and social relationships can affect learning resilience by having an effect on individual characteristics (academic self-efficacy, perceived campus belonging) of students, which keeps changing the level of academic engagement^21–23^. As a result, to increase the level of academic engagement and academic performance of students, schools need to pay attention to developing an external environment that is able to promote learning resilience. This would imply fulfilling essential psychological requirements of students, changing learning behaviors, and encouraging learning motivation of students by influencing individual characteristics, i.e., academic self-efficacy and perceived campus belonging.

### Triadic interaction determinism

Triadic interaction determinism is a central tenet of social cognitive theory which states that individual characteristics, behavioral determinants, and environmental determinants are mutually exclusive but mutually interdependent to determine behavior, not controlled by any one determinant^2,19^. In the present study, learning environment, learning climate, and social relationships are conceptualized as environmental determinants,academic self-efficacy and perceived campus belonging are conceptualized as proximal individual determinants; and learning resilience is conceptualized as the behavioral outcome. This classification follows the logic of social cognitive theory and triadic reciprocal determinism, and it provides the theoretical basis for the subsequent SEM specification.

Triadic interactionism posits that individual characteristics, environmental conditions, and behavioral performance are not related to each other in a simplistic manner, but instead there is a multidirectional, active and cyclical triangle of relations between them^24,25^. Learners are more prone to demonstrate high learning resilience when placed in situations where the activities in the learning process can be seen as meaningful, encounter moderate difficulties and receive assistance by a variety of sources^6,26^. This research thus considers the concept of learning resilience as the main outcome variable which is going to be measured on the effect of the environment on individual behavioral reactions based on the triadic interactionist theory. The goal is to investigate and affirm students’ capability to overcome failures and resist pressure in the learning process and, consequently, disclose the very essence of the mechanisms of improving learning resilience. Through this theoretical framework, we can more accurately grasp how to optimize learning environments and stimulate individual traits to shape students’ learning resilience, addressing core issues in educational practice such as insufficient learning motivation and low engagement. Accordingly, the SEM was constructed to reflect this theoretical structure rather than to test isolated correlations. Specifically, learning environment, learning climate, and social relationships were modeled as distal contextual perceptions,academic self-efficacy and perceived campus belonging were modeled as proximal psychological mechanisms; and learning resilience was modeled as the outcome. Direct paths from the three contextual variables to learning resilience were retained to examine whether external context had residual explanatory value beyond the mediators, whereas indirect paths were specified to test the theory-based expectation that external support is translated into resilience primarily through students’ self-beliefs and relational-emotional evaluations.

In this study, this theoretical framework is interpreted as implying that external context may function more as a distal condition than as an immediate determinant of learning resilience. Accordingly, learning environment, learning climate, and social relationships were modeled as contextual antecedents, whereas academic self-efficacy and perceived campus belonging were modeled as more proximal psychological mechanisms. On this basis, direct paths from contextual variables to learning resilience were retained not because strong direct effects were assumed, but to examine whether external context had explanatory value beyond these mediators.

### Academic self-efficacy

Academic self-efficacy refers to an individual’s subjective judgment and belief regarding their ability to successfully complete a task. It influences an individual’s choice of behavior, level of effort, and persistence^4,18,27^. Bandura proposed that academic self-efficacy primarily develops through four sources: the most direct influence of one’s own success or failure in practice, the most relevant success or failure experiences of others similar to oneself; the most empathetic encouragement, advice, and evaluations from others; and one’s management of emotions-particularly the control of negative emotions and self-regulation of psychological imbalance^16,28,29^.

Academic self-efficacy is not a static personality trait. It changes over time due to individual experiences and shifts in the environment^30,31^. One successful experience can quickly boost one academic self-efficacy, which can surpass one real level of capability. A sequence of failures, however, might result in serious frustration and self-doubt, leading people to underestimate their skills significantly or even incorrectly evaluate their skills. Students might show great learning resilience and enjoy the situation when experiencing high academic self-efficacy and facing unexpected challenges or obstacles^4,27^. So, this research considers academic self-efficacy as the emotional basis and takes the concept of learning resilience as the final variable. The purpose of this research is to investigate in detail and confirm students adaptive and regulatory actions, persistence, and negative emotion regulation in the learning process, identifying the underlying principles that can be used to improve the learning resilience. With the help of this framework, we will be able to obtain a more precise vision of what needs to be done to create optimal learning conditions, academic climates, and interpersonal relationships to trigger academic self-efficacy, thus develop students academic resilience, and solve fundamental problems like lack of motivation to learn and low involvement.

## Objectives and hypotheses

### Research objectives

**Fig. 1:**
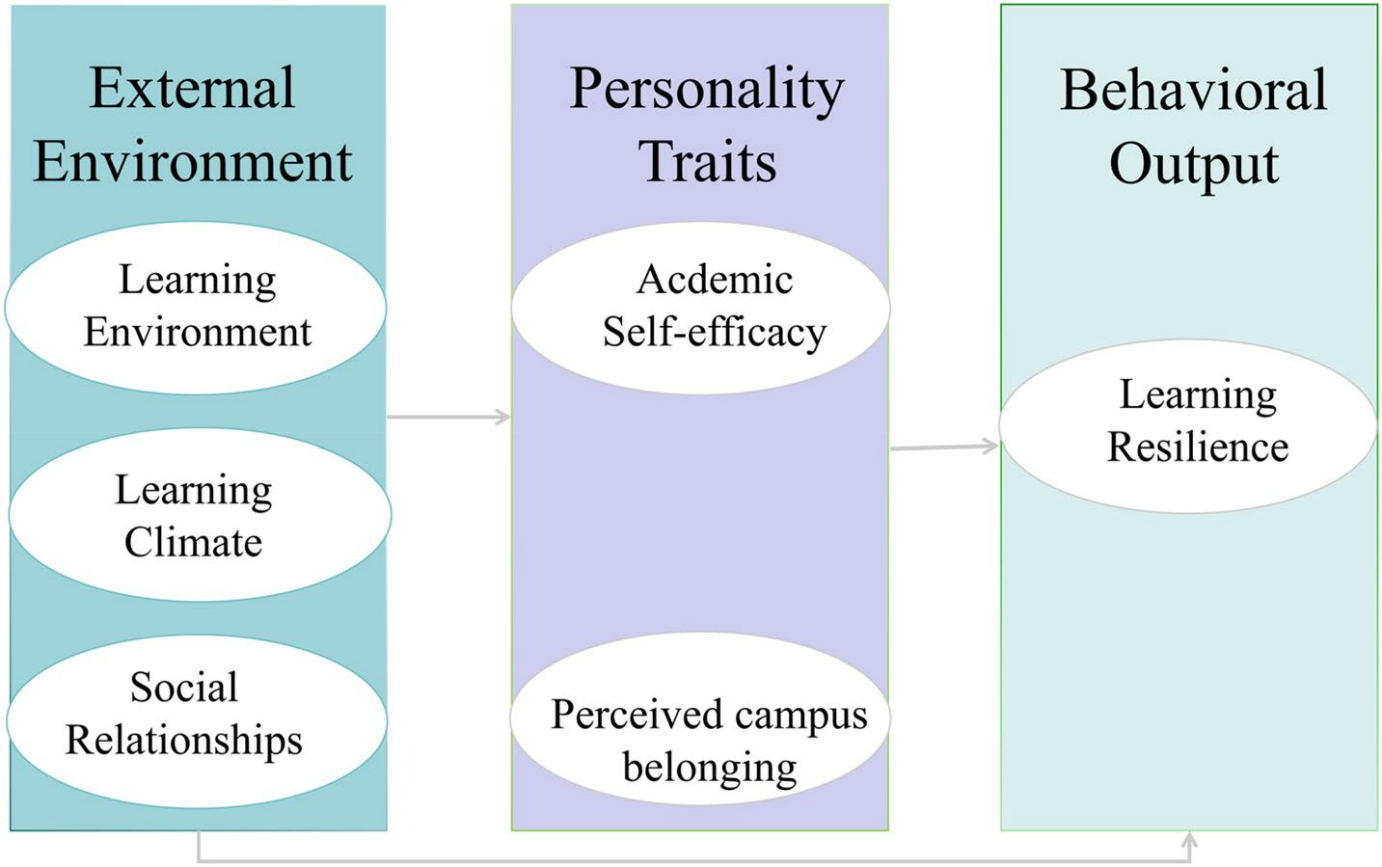
Conceptual model of “external context-individual characteristics-learning resilience”.

The purpose of this paper is to examine, under the framework of social cognitive theory, triadic interactionism as well as the bridging function of academic self-efficacy, the extent to which college students perceptions of learning environment, academic climate and social connection are predictive of their learning resilience; if learning academic self-efficacy and perceived campus belonging could play an important role as mediators between perceived external learning environment and learning resilience; and which external contextual variables show the strongest statistical associations with academic self-efficacy and learning resilience. To sum up, the authors methodologically discuss the generative processes of learning resilience of college students in various external learning conditions via an environment-individual-behavior logical chain, especially confirming the mediating link of academic self-efficacy in the context of external ecological assistance and resilience behavior. Structural equation modeling (SEM) is used in this research by showing how learning environment, academic climate and social relationships interact to support the process of building students self-belief. The SEM framework allows us to examine how learning environment, learning climate, and social relationships are statistically related to students’ self-beliefs and resilience-related outcomes within one theoretically specified model. As it has been shown in Fig. 1.

Guided by social cognitive theory and triadic reciprocal determinism, this study conceptualizes learning environment, learning climate, and social relationships as external contextual perceptions, academic self-efficacy and perceived campus belonging as proximal individual mechanisms, and learning resilience as the outcome variable. On this basis, the SEM was designed to test three theoretically grounded questions: whether external contextual perceptions are associated with academic self-efficacy and perceived campus belonging, whether these two individual factors are associated with learning resilience, and whether they mediate the relationships between external context and learning resilience. SEM was selected because it allows the simultaneous estimation of multiple direct and indirect paths within one theoretically specified model. The conceptual model is shown in Fig. 1.

In order to further clarify the connection between particular extrinsic environmental factors and personal traits, and the interaction between such variables in determining the learning resilience outcome, researchers categorized indicators in all dimensions (refer to Table 1).

**Table 1:** Item dimensions and indicator codes.

Dimension	Indicator number	Title statement
Learning Environment (LE)	LE1	The university’s administrative and support services (e.g., course registration, dormitory services, and technical support) effectively support my learning
	LE2	The university’s course arrangements and academic policies are fair to students
	LE3	I believe that the university provides sufficient learning resources (e.g., libraries, study rooms, and internet access)
	LE4	I feel that the campus environment stimulates my motivation to learn
	LE5	I believe that the university’s learning environment is safe, clean, and conducive to focused study
Learning Climate (LC)	LC1	In class, teachers encourage us to express our own views and ideas
	LC2	The learning climate in my class or course is positive, and my classmates take their studies seriously
	LC3	I often receive helpful feedback from teachers that helps me improve my learning
	LC4	I feel that teachers genuinely care about students’ learning and development
	LC5	The assignments and tasks provided by teachers are appropriately challenging and stimulate my interest in learning
Social Relationships (SR)	SR1	I have positive relationships with my classmates, and we support and help one another
	SR2	I have good communication and interaction with my teachers
	SR3	At school, I have people whom I can trust and confide in
	SR4	I feel accepted and respected on campus
	SR5	When I encounter learning difficulties, I can obtain timely help from my peers
Academic Self-efficacy (ASE)	ASE1	I am confident that I can master the most essential and challenging knowledge in my current major courses
	ASE2	I am certain that I possess the intelligence and ability required to achieve excellent academic performance
	ASE3	Even when the learning content is highly complex, I believe that I can eventually understand and master it well
	ASE4	Even when academic tasks are very demanding, I believe that I can complete them on time through effort
	ASE5	When I encounter bottlenecks or setbacks in learning, I am confident that I can find solutions
	ASE6	I believe that I can flexibly adjust my learning strategies to cope with the challenges of different courses
Perceived campus belonging (PCB)	PCB1	I feel that I am truly a part of this university, have a strong sense of presence here, and am proud of it
	PCB2	I identify with this university’s campus culture, academic culture, and educational philosophy
	PCB3	Even if I could choose again, I would still choose the university I am currently attending
	PCB4	I believe that the overall campus atmosphere is positive and inclusive, making it comfortable to study here
	PCB5	I believe that teachers and students at this university respect one another, are friendly, and maintain harmonious relationships
	PCB6	I believe that this university has a strong academic atmosphere and that my views are respected and supported
Learning Resilience (LR)	LR1	When I encounter setbacks in my studies, such as failing an exam, I will redouble my efforts
	LR2	Even if I encounter extremely great difficulties in my study, I will not easily have the thought of giving up
	LR3	I can persevere in solving those academic problems that seem extremely complex
	LR4	If a certain learning method proves ineffective, I will proactively seek new strategies or approaches
	LR5	I can calmly analyze the reasons for my academic failure and draw lessons from it
	LR6	In the face of sudden changes in the learning environment, I can quickly adjust my mindset to get into the right state
	LR7	Even when facing huge pressure from the final exams, I can still keep my emotions stable and focus on my studies
	LR8	I will not deny my value or ability just because of a temporary decline in my academic performance
	LR9	When my study progress lags behind, I can overcome the anxiety and make a remedial plan

### Research hypotheses

**Fig. 2:**
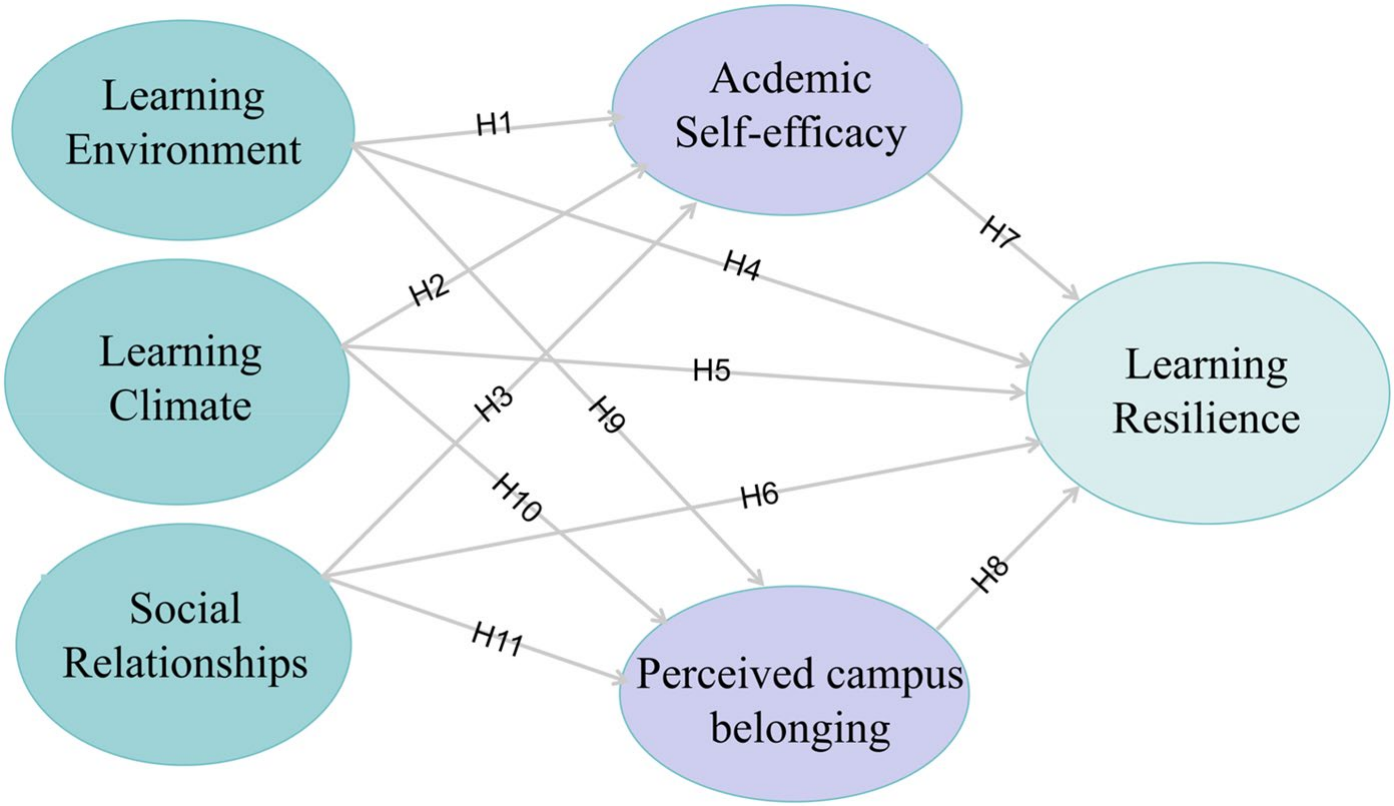
Conceptual model.

This study proposes that external environmental support, including learning environment, learning climate, and social relationships, may be positively associated with students’ learning resilience through the mediating roles of academic self-efficacy and perceived campus belonging. It also promotes the learning involvement of students so as to ensure they attain important academic achievements. According to the available literature, it is evident that students being exposed to an external learning environment that is resource-rich, has a strong learning climate, and positive social relationships show improved proactive and persistence in their studies. Their enthusiasm towards learning remains constant throughout the learning process, they regulate their emotions in a positive way whenever they face academic challenge and obstacles, and they eventually have more composed behaviors in learning and achieve higher academic results^16,18,29,30^. Then this study assumes that an excellent quality external environment has provided students with necessary learning conditions, a comfortable learning environment and positive social relations. These factors might trigger their interest and motivation in learning on an intrinsic level and change learning to be an unconscious, spontaneous and autonomous act which leads to positive learning results. Nevertheless, other studies based exclusively on internal factors do not completely represent the complex interplay between the individual features and learning resilience^13,32^. Thus, in this study, the independent variables are the external environment (learning environment, learning atmosphere, social relationships) and the mediating variables are individual characteristics in the learning ecosystem (academic self-efficacy, perceived campus belonging), and the outcome variables are learning resilience (persistence, self-reflection and adaptability, negative emotion regulation). It explores the power of these three sets of variables and their particular indicators over each other and their mutual interactions. The research especially investigates the predictive value of mediating variables upon outcome variables and if different levels of learning resilience affect perceptions of academic engagement and academic success. To sum up, the results will enable educational administrators to learn more about the intrinsic relationship between the concepts of learning environment-individual characteristics-learning resilience, making clear why learning resilience matters in terms of academic engagement and academic performance. These findings may help educational administrators better understand the relationships among learning environment, individual characteristics, and learning resilience, and may offer tentative implications for teaching practice and student support. In light of this, the researcher has mapped the relationships among the six variables involved in the study, as shown in Fig. 2.

#### Learning environment perception and academic self-efficacy, perceived campus belonging

Existing research indicates that when students perceive external environmental support, they develop a strong motivation to learn^33,34^. This positive external feedback not only safeguards learning outcomes but also indirectly enhances intrinsic academic self-efficacy and perceived campus belonging, thereby boosting learning resilience to overcome and navigate academic adversities and challenges^35–37^. Numerous studies indicate that a positive learning environment and climate can stimulate students’ curiosity and desire to explore, enhancing their academic self-efficacy in simple terms, igniting their passion for learning and boosting their confidence^38–40^. In general, the more advantageous the learning environment and the richer the learning resources available to students, the more confident and composed they become in their studies, ultimately achieving higher academic performance^41–44^. Based on this, we hypothesize that:

##### H1

Perceived learning environment support positively predicts academic self-efficacy.

##### H2

Perceived learning environment support positively predicts perceived campus belonging.

#### Learning climate, social relationships, and academic self-efficacy and perceived campus belonging

A positive learning environment and active social interactions enable students to feel supported and encouraged by others during their studies, thereby enhancing individual learning confidence and further fostering strong learning resilience^45^. The learning climate, sense of belonging and conviction within the learning community serve as crucial pillars for academic self-efficacy and perceived campus belonging^46^. Given this, we hypothesize that:

##### H3

Learning climate positively predicts academic self-efficacy.

##### H4

Learning climate positively predicts perceived campus belonging.

##### H5

Positive social relationships positively predict academic self-efficacy.

##### H6

Positive social relationships positively predict perceived campus belonging.

#### Personality traits (academic self-efficacy, perceived campus belonging) perception and learning resilience

According to existing research, both academic self-efficacy and perceived campus belonging can promote students to develop strong learning resilience^47,48^. Learning resilience, as an underlying force driving student learning behaviors, has a positive effect on promoting academic engagement^49^. Based on this, we hypothesize that underlying individual traits (academic self-efficacy, perceived campus belonging) exert a significant positive influence on students’ learning resilience. Specifically:

##### H7

Academic self-efficacy positively predicts learning resilience.

##### H8

Perceived campus belonging positively predicts learning resilience.

#### External environment (learning environment, learning climate, social relationships) support and learning resilience

Previous studies have confirmed that external learning environments (learning environment, learning climate, social relationships) exert a significant influence on students’ learning resilience^32,50^. External environment support primarily encompasses perceptions of the learning environment, learning climate, and social relationships, all of which contribute to varying degrees to the development of students’ learning resilience. Based on this, we hypothesize that:

##### H9

A positive learning environment positively predicts learning resilience.

##### H10

A positive learning climate positively predicts learning resilience.

##### H11

Positive social relationships positively predict learning resilience.

#### Mediating mechanisms between external environment and learning resilience

Based on social cognitive theory’s triadic interactionism, environmental factors do not directly determine behavioral outcomes but exert indirect influence through individuals’ cognitive and emotional processes (i.e., “subjective factors”). Therefore, the direct paths from external context to learning resilience were retained in the SEM primarily to test whether any residual direct association remained beyond the mediators, rather than to assume that strong direct effects would necessarily be supported. This study posits that academic self-efficacy and perceived campus belonging play a crucial mediating role between external environmental support (learning environment, learning climate, social relationships) and learning resilience.

##### Mediating role hypothesis of academic self-efficacy

Academic self-efficacy is an individual’s belief in their ability to accomplish specific tasks. According to Bandura’s theory, supportive feedback from the external environment, such as encouragement from teachers, mutual assistance from peers, and readily available learning resources, serves as a crucial source for enhancing an individual’s academic self-efficacy. Existing research indicates that positive teacher-student relationships, as a key environmental factor, can effectively enhance students’ learning motivation and academic self-efficacy^3^. When college students perceive a positive learning environment and strong interpersonal relationships, they reinforce their belief in “I can do it” through vicarious experiences and verbal persuasion. The improved academic self-efficacy will be a very effective type of internal psychological capital that allows students to demonstrate increased resilience and persistence in the face of academic challenges. Investigations conducted by Huang and Liu^4,6^ have established a high correlation between academic self-efficacy and learning resilience with academic self-efficacy acting as the emotional base to bring out resilient responses. Additionally, Jiao et al.^6^ also found that academic self-efficacy plays a crucial mediating role between motivation and psychological resilience. In other words, the external environment must first be transformed into internal confidence before resilience can ultimately be enhanced. Based on this, the following hypothesis is proposed:

###### H12

Academic self-efficacy mediates the relationship between learning environment and “learning resilience.”

###### H13

Academic self-efficacy mediates the relationship between learning climate and learning resilience.”

###### H14

Aacademic self-efficacy mediates the relationship between social relationships and learning resilience.

##### Mediating role hypothesis of perceived campus belonging

Perceived campus belonging refers to the degree to which students psychologically feel accepted, respected, and supported by the school community. According to self-determination theory, this sense of belonging serves as a crucial emotional prerequisite for student engagement in learning activities. A high-quality learning environment and supportive social network directly fulfill students’ fundamental psychological needs, thereby fostering perceived campus belonging. As shown by Shao et al.^9^, peer relations and other environmental conditions play a significant role in determining the psychological welfare and academic achievements of students through chain-mediated mechanisms. The feeling of safety in the face of academic failures is very helpful when students have a strong perceived campus belonging^15,27,51^. It states that the satisfaction of fundamental psychological requirements, including belonging, may foster learning involvement along the path of resilience. More significantly, the study of Mtshweni^51^ confirms the mediation of perceived campus belonging in the link between emotional adjustment and academic persistence (an indicator of resilience) in a chain form. Thus, the development of learning resilience is also promoted indirectly by an external environmental support which enhances students psychological sense of belonging. On this basis, the hypothesis formulated is:

###### H15

Perceived campus belonging mediates the relationship between learning environment and learning resilience.

###### H16

Perceived campus belonging mediates the relationship between learning climate and learning resilience.

###### H17

Perceived campus belonging mediates the relationship between social relationships and learning resilience.

## Research methods

### Scale development

To collect comprehensive data from students at Zhejiang Normal University, we designed a structured questionnaire consisting of two parts: demographic questions and 36 substantive items measuring the six latent constructs in this study. Specifically, the measurement section included 15 items assessing external contextual perceptions^52^ (learning environment, learning climate, and social relationships), 12 items assessing academic self-efficacy and perceived campus belonging^53,54^, and 9 items assessing learning resilience^51^.

All measurement items were rated on a Likert-type scale. The remaining items focus on examining whether external environment variables are associated with learning resilience through academic self-efficacy and perceived campus belonging. These relationships may also be relevant to students’ academic engagement and performance, although such implications should not be interpreted causally in the present cross-sectional design. This, in turn, may further drive college students’ academic engagement and help them achieve positive academic performance. To ensure reliability and content validity, questionnaire items were developed primarily by adapting existing measurement scales and underwent rigorous validation procedures. Regarding the issue of consistency between the Chinese and English questionnaires, the researchers first translated the questionnaire from English into Chinese, then back-translated it into English to confirm conceptual equivalence. The final Chinese translation was reviewed and calibrated by two experts in the relevant field.

### Data collection

In January 2026, researchers employed a non-probability snowball sampling method to conduct an online electronic questionnaire survey using the domestic Questionnaire Star platform. Regarding questionnaire distribution, the researchers contacted faculty members at Zhejiang Normal University via WeChat and requested their assistance in forwarding the electronic survey to university students. The total number of questionnaires that have been distributed and collected is 620. When submissions with abnormalities and responses that are filled out in less than 60 s are removed, the remaining 515 valid questionnaires are obtained, which gives a response rate of 83 percent.

The privacy and security of data and information provided by respondents were safeguarded through various measures undertaken by researchers to guarantee the confidentiality of information. Researchers, for example, have anonymized all the responses and encoded and encrypted the data. According to the ethical guidelines of conducting questionnaire surveys, researchers provided a short description of the aim of the study and listed the questions to be asked to respondents before starting the survey. The researchers followed the principle of voluntary involvement of respondents after handing out the questionnaires and made it clear that the data would be used in academic research only to enable the smooth conduct of the survey. All methods were carried out in accordance with relevant guidelines and regulations. All experimental protocols were approved by the institutional ethics committee of Zhejiang Normal University (Review Number: ZSRT2026077). Informed consent was obtained from all subjects involved in the study. Because the data were collected at a single time point in January 2026, the SEM results should be interpreted as theory-consistent associations rather than causal effects.

## Results

According to Table 2, the demographic information shows that of the 515 individuals, 240 were men (46.60 percent) and 275 were women (53.40 percent), which is a fairly equal share of males and females. First-year students made up 54.86 percent of the total number of respondents in the questionnaire survey, with second- and third-year students making up 44.47 percent of all respondents.

**Table 2:** Demographics (N = 515).

Variable	Frequency	Percentage %
Gender		
Male	240	46.6
Female	275	53.4
Grade		
Freshman	281	54.56
Sophomore	175	33.98
Junior	54	10.49
Senior	5	00.77
Weekly Learning Hours		
≤ 5 h	103	20
5 h-10 h(excluded 10 h)	191	37.09
10 h-15 h(excluded 15 h)	106	20.58
15-20 h(excluded 20 h)	53	10.29
≥ 20 h	62	12.04
Primary learning methods		
Self-directed learning	492	95.53
Group learning	78	15.15
Classroom lectures	292	56.7
Online learning	230	44.66
Tutoring classes, etc	17	3.3
Other (online courses)	10	1.94
Whether or not to join school clubs		
Yes	303	58.83
No	212	41.17

### Measurement model

Table 1 presents the full set of measurement items for all six constructs, including the learning resilience scale, which was omitted in the previous version of the manuscript. As shown in Table 3, the Cronbach’s alpha coefficients and composite reliability for all constructs met the requirements, with values > 0.70.Then we employed the four tests recommended by Hair, J. F. et al. to examine convergent validity and discriminant validity^55^.

**Table 3:** Construct measurement and confirmatory factor analysis.

Dimension	Indicator	Mean	Standard deviation	Factor loadings	Cronbach’s alpha	CR	AVE
LE	LE1	4.458	0.768	0.901	0.937	0.952	0.798
	LE2	4.458	0.752	0.899			
	LE3	4.277	0.914	0.892			
	LE4	4.227	0.946	0.863			
	LE5	4.301	0.856	0.911			
LC	LC1	4.323	0.827	0.907	0.934	0.953	0.836
	LC2	4.269	0.848	0.933			
	LC3	4.237	0.871	0.919			
	LC4	4.253	0.860	0.897			
SR	SR1	4.410	0.815	0.916	0.946	0.959	0.823
	SR2	4.448	0.759	0.919			
	SR3	4.380	0.823	0.919			
	SR4	4.371	0.822	0.907			
	SR5	4.285	0.952	0.875			
ASE	ASE1	4.084	0.883	0.884	0.960	0.968	0.833
	ASE2	4.110	0.902	0.890			
	ASE3	4.157	0.873	0.941			
	ASE4	4.173	0.882	0.915			
	ASE5	4.179	0.859	0.928			
	ASE6	4.175	0.866	0.917			
PCB	PCB1	4.030	0.962	0.846	0.952	0.962	0.807
	PCB2	4.175	0.903	0.912			
	PCB3	4.000	1.037	0.875			
	PCB4	4.237	0.871	0.932			
	PCB5	4.297	0.804	0.896			
	PCB6	4.281	0.841	0.928			

Test 1: As shown in Table 4, the mean variance extracted values for all constructs exceeded 0.50, surpassing the threshold recommended by Hair et al.^55^.

**Table 4:** HTMT validity analysis.

	PCB	LC	LE	LR	ASE	SR
PCB						
LC	0.829					
LE	0.815	0.868				
LR	0.900	0.791	0.761			
ASE	0.842	0.735	0.689	0.894		
SR	0.839	0.895	0.857	0.821	0.747	

Test 2: As shown in Table 4, the square roots of the AVE values for each construct exceeded their respective off-diagonal correlations, which generally aligns with the recommendations of Hair et al.^55^.

Test 3: As shown in Table 3, the loadings for each item exceeded 0.70, consistent with the findings reported by James F. Hair Jr. et al.

Test 4: Based on the method proposed by Hair et al.^55^, we evaluated the heterothetic-to-homothetic ratio (HTMT). This is achieved by dividing the average correlation between different constructs by the average correlation among indicators within the same construct. Upon examination, the HTMT values for all established variables fell below the 0.90 threshold recommended by Hair et al. (In most cases, an HTMT value below 0.90 is acceptable for establishing discriminant validity, particularly when constructs are closely related—such as emotional and cognitive dimensions—where the threshold may be relaxed to 0.90).In summary, these results indicate that the measurement exhibits satisfactory convergent validity and discriminant validity.

Although the mean HTMT scores for some constructs approached or reached the lenient threshold of 0.90 (e.g., perceived campus belonging and learning resilience), this study employed the HTMT Inference method to rigorously validate empirical differences between constructs.Results from 5000 bootstrap resampling analyses indicate that the upper bounds of the 95% bias-corrected confidence intervals for HTMT values across all constructs are all less than 1 (with the highest value being 0.935), and none of the confidence intervals include 1. This provides strong evidence that the model in this study possesses good discriminant validity.

### Hypothesis testing

We employed a self-service approach to test the hypotheses of the structural equation model. First, we calculated the variance inflation factor (VIF) using Smart-PLS3.None of the VIF values exceeded 5, indicating no multicollinearity issues. The R^2^values for academic self-efficacy, perceived campus belonging, and learning resilience were 0.544, 0.700, and 0.825. However, the comparatively high R^2^ for learning resilience should be interpreted in light of the fact that learning resilience was directly predicted by academic self-efficacy and perceived campus belonging, whereas the direct effects of some external contextual variables were weak or non-significant. Based on the revised hypothesis numbering system, Table 5 presents the hypothesis-testing results in a clearer one-to-one correspondence between hypotheses and structural paths. Specifically, H2–H8, H11, and H13–H17 were supported, whereas H1, H9, H10, and H12 were not supported at the conventional 0.05 level. More specifically, learning environment significantly predicted perceived campus belonging, but its paths to academic self-efficacy and learning resilience did not reach statistical significance. Learning climate significantly predicted academic self-efficacy and perceived campus belonging, but its direct path to learning resilience was not significant. Social relationships significantly predicted academic self-efficacy, perceived campus belonging, and learning resilience. In addition, both academic self-efficacy and perceived campus belonging significantly predicted learning resilience. Regarding the mediation hypotheses, the indirect paths through academic self-efficacy were supported for learning climate and social relationships, but not for learning environment, whereas all three indirect paths through perceived campus belonging were supported.

**Table 5:** Hypothesis testing.

Hypothesis	Path coefficient	Standard deviation	T-value	*P* value	Result
LC—> LR	0.017	0.051	0.327	0.744	Not accepted
LC—> ASE	0.277	0.072	3.857	< 0.001***	Accepted
LC—> PCB	0.261	0.065	4.009	< 0.001***	Accepted
LE—> LR	0.026	0.043	0.598	0.550	Not accepted
LE—> ASE	0.123	0.068	1.808	0.071	Not accepted
LE—> PCB	0.266	0.058	4.565	< 0.001***	Accepted
ASE—> LR	0.430	0.058	7.453	< 0.001***	Accepted
PCB- > LR	0.352	0.068	5.140	< 0.001***	Accepted
SR—> LR	0.158	0.054	2.930	< 0.05*	Accepted
SR—> ASE	0.380	0.078	4.866	< 0.001***	Accepted
SR—> PCB	0.363	0.065	5.623	< 0.001***	Accepted
LC- > ASE- > LR	0.119	0.036	3.276	< 0.01**	Accepted
LE- > ASE- > LR	0.053	0.030	1.758	0.079	Not accepted
SR- > ASE- > LR	0.163	0.040	4.103	< 0.001***	Accepted
LC- > PCB- > LR	0.092	0.028	3.250	< 0.01**	Accepted
LE- > PCB- > LR	0.094	0.027	3.421	< 0.01**	Accepted
SR- > PCB- > LR	0.128	0.035	3.639	< 0.001***	Accepted

Note: *P* < 0.05, Significant influence*; *P* < 0.01, Representative influence is relatively significant**; *P* < 0.001, The impact is highly significant***

As shown in Table 5, the results of testing the hypotheses regarding the correlations among factors influencing learning resilience are as follows: academic self-efficacy and perceived campus belonging exhibit highly significant correlations with learning resilience at the *P* < 0.001 level; learning climate shows less significant correlation with learning resilience at the *P* < 0.05 level; Social relationships and learning resilience exhibit a significant correlation at the *P* < 0.01 level; however, the relationship between learning environment and learning resilience is less significant at the *P* < 0.05 level. It follows that the mediating variables academic self-efficacy and perceived campus belonging are positively correlated with learning resilience. Empirical evidence confirms that these two factors can predict learning outcomes resulting from enhanced learning resilience. As a single factor representing external environmental support, the learning environment has a weak correlation with the development of learning resilience. However, both the learning environment and social relationships exert positive influences on the development of learning resilience at the individual level. This demonstrates that emotional and cognitive factors can provide sustained, reflective, and adaptive motivation for emotional management among college students at the psychological level.

**Fig. 3:**
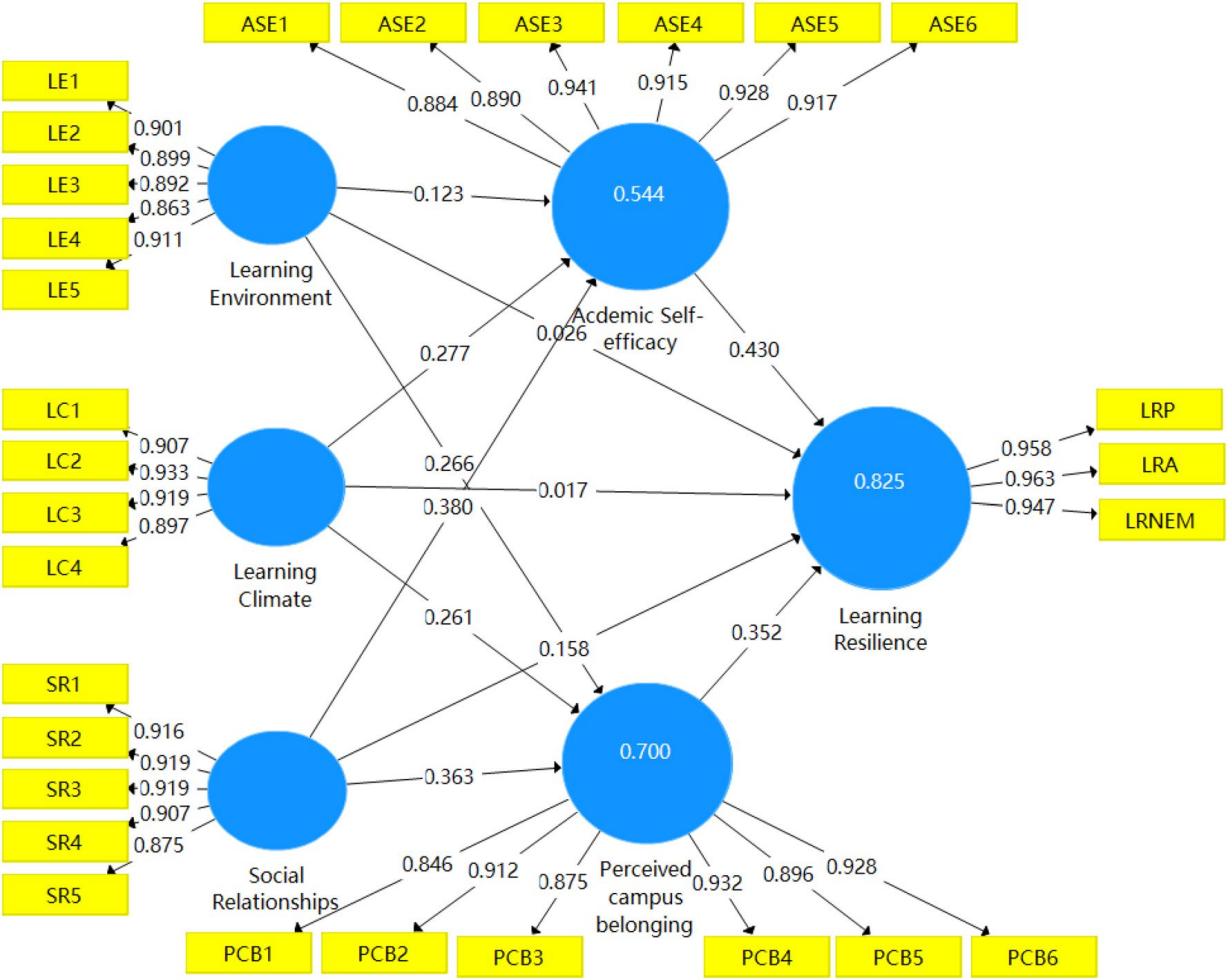
Path coefficients and R^2^.

The path analysis results in Fig. 3 support most of the hypothesized relationships in our model, but some paths did not reach the level of significance. Perceived learning environment support showed positive but statistically non-significant associations with academic self-efficacy (*β* = 0.123,* p* = 0.071) and learning resilience (*β* = 0.026, *p* = 0.550), while showing a significant positive association with perceived campus belonging (*β* = 0.266, *p* < 0.001). The learning climate exerted highly significant effects on both academic self-efficacy (*β* = 0.227, *P* < 0.001) and perceived campus belonging(*β* = 0.261, *P* < 0.001), but its correlation with learning resilience was not significant (*β* = 0.017, *P* > 0.05).Social interaction exerts a highly significant positive influence on academic self-efficacy (*β* = 0.380, *P* < 0.001) and perceived campus belonging(*β* = 0.363, *P* < 0.001), and a relatively significant influence on learning resilience (*β* = 0.158, *P* < 0.01). Academic self-efficacy (*β* = 0.430, *P* < 0.001) and perceived campus belonging (*β* = 0.352,* P* < 0.001) exerted highly significant effects on learning resilience. Acting as a mediating bridge, academic self-efficacy significantly influenced the relationship between learning climate and learning resilience (*β* = 0.053, *P* < 0.001), and between social interaction and learning resilience (*β* = 0.163, *P* < 0.001). Perceived campus belonging, as a mediating factor, significantly influenced the relationships between learning climate and learning resilience (*β* = 0.092, *P* < 0.001), learning environment and learning resilience (*β* = 0.094, *P* < 0.001), and social interaction and learning resilience (*β* = 0.128, *P* < 0.001), By contrast, the indirect path from learning environment to learning resilience through academic self-efficacy was positive but did not reach the 0.05 level of significance (*β* = 0.053, *p* = 0.079), whereas the indirect path through perceived campus belonging was statistically significant (*β* = 0.094, *p* < 0.01).This finding validates the mediating role of the intervening variables (academic self-efficacy and perceived campus belonging) as crucial bridges linking the independent variables (learning environment, learning climate, and social relationships) to the outcome variable (learning resilience). It effectively mediates the effect of independent variables on the outcome variable, providing educational administrators with key evidence-based references for intervening in learning resilience and mobilizing academic engagement strategies.

These findings indicate that the model does not support strong direct effects of learning environment or learning climate on learning resilience in the present sample. Instead, the statistically strongest paths are located at the level of academic self-efficacy and perceived campus belonging, together with several indirect paths linking contextual variables to learning resilience through these two mediators. This pattern suggests that learning resilience may be more proximally associated with internal psychological mechanisms than with external context alone. At the same time, the significant but modest path from social relationships to learning resilience implies that some contextual dimensions may retain limited direct explanatory value. Therefore, the results are more consistent with a proximal-mechanism interpretation than with a strong direct-context model. Meanwhile, the data in Table 5 indicate that individual traits positively influence students’ learning resilience through a mediating role and are associated with learning engagement. These results indicate that the direct effects of learning environment and learning climate on learning resilience were not supported in the present sample and should therefore be interpreted cautiously in theoretical terms.

### Discussion

This study, grounded in social cognitive theory, triadic interaction theory, and the concept of learning resilience, delves into the mechanisms by which individual characteristics (academic self-efficacy and perceived campus belonging) are formed among college students across different external learning environments, and examines the pathways through which these characteristics influence their levels of learning resilience. By constructing and validating a structural model of “external environment- personality traits-learning resilience,” we revealed the affective orientation and situational basis of college students’ personal traits. In terms of specific research findings, external learning environments, particularly learning climate support and social relationship support, were found to be positively associated with students’ academic self-efficacy and perceived campus belonging. These psychological factors were, in turn, positively associated with learning resilience and may help explain differences in how students respond to academic adversity. This key conclusion is strongly supported by the empirical data in this study and partially aligns with relevant findings in the existing literature. From an academic perspective, this clearly demonstrates that in the study of learning environments, the learning climate and social relationships as significant emotional factors within the external environment, play a substantial role in enhancing students’ academic self-efficacy and perceived campus belonging. This highlights the mediating effect of personal characteristics (academic self-efficacy and perceived campus belonging) on learning resilience. Importantly, the weak direct paths from learning environment and learning climate to learning resilience should not be framed as an unexpected discovery, but rather as unsupported direct-effect hypotheses in the present sample. Accordingly, the results are better interpreted as indicating that contextual factors may operate more indirectly than originally phrased in the Introduction.

An important theoretical implication of these results is that the model is better interpreted as a proximal psychological mechanism model than as a strong direct-effect model of external context. Although the original framework assumed that external learning contexts would contribute to learning resilience, the empirical results indicate that this contribution is expressed more clearly through academic self-efficacy and perceived campus belonging than through direct contextual effects. In particular, the non-significant paths from learning environment and learning climate to learning resilience, together with the comparatively stronger paths from academic self-efficacy and perceived campus belonging to learning resilience, suggest that resilience in the present sample is more immediately related to students’ self-beliefs and relational-emotional evaluations. Rather than treating this pattern as a failure of the broader theoretical framework, we interpret it as evidence that external context may operate as a distal antecedent whose effects are filtered through more proximal psychological processes. At the same time, the weak direct effects also suggest that future models should incorporate additional factors, such as self-regulation, motivation, emotional adjustment, or prior academic experience, in order to explain learning resilience more comprehensively.

Therefore, by adjusting academic self-efficacy and perceived campus belonging, the sustained impact of external learning environments on learning resilience and engagement can be enhanced. A more supportive external learning context may be associated with learning resilience through its links with academic self-efficacy and perceived campus belonging, thereby increasing college students’ learning confidence and sense of accomplishment. Evidently, identifying the mediating roles of academic self-efficacy and perceived campus belonging in the associations between external learning contexts and learning resilience may offer educational administrators some tentative practical insights. However, these implications should be interpreted cautiously, because the direct effects of learning environment and learning climate on learning resilience were not supported, and the statistically significant indirect effects were modest in magnitude. For instance, the findings tentatively suggest that educational administrators and teachers may consider creating more supportive classroom climates, strengthening constructive feedback practices, encouraging peer collaboration, and fostering inclusive teacher–student and peer relationships. These practices may be relevant because they are associated with academic self-efficacy and perceived campus belonging, which in turn were associated with learning resilience in the present sample. Nevertheless, given the weak direct contextual effects and the modest size of several indirect effects, these suggestions should be regarded as cautious practice implications rather than strong intervention prescriptions.

The present study offers a more differentiated account of how social relationships and learning climate may be associated with learning resilience through personal factors such as academic self-efficacy and perceived campus belonging, although the practical magnitude of these pathways should not be overstated. In particular, studies have shown that the resilience of students to learn is strongly associated with their learning environment and social relationships, and it also depends greatly on their individual characteristics. Of them, student academic self-efficacy is considered as an important emotional basis of learning resilience and is closely aligned with the underlying principles of positive psychology and education. Academic self-efficacy is derived out of students acknowledging their own capacity to learn and their potential, based on a real love of the learning process and consistent efforts towards gaining knowledge. It is extremely motivating in encouraging students to participate in learning tasks and bravely conquer different hardships and obstacles. A feeling of membership of campus and social relationships contributes to the stability and consistency of academic self-efficacy, which has a strong positive impact on learning resilience. As an example, properly balanced external rewards, support, and recognition, i.e., as manifestations of perceived campus belonging, may increase students confidence and sense of achievement to some extent and thus successfully motivate them to develop academic self-efficacy. Students who have strong social ties have rich learning resources and are well-supported. Their peer support allows them to get better acquainted with course material, evaluate their personal situations, and define their learning aims. This then leads to having the courage to face challenges and improves their capacity to handle academic problems through sustained efforts. Hence, the results provide partial support for the view that individual psychological mechanisms mediate the associations between external context and learning resilience, although the strength of these pathways differs across contextual dimensions. The result confirms the findings of past researches and adds to their results using empirical data.

From a practical perspective, the model offers tentative insights for educational managers regarding the psychological and relational factors associated with learning resilience. Teachers may use this model by creating a favorable learning climate, promoting a high level of cooperation and communication between students, and developing a friendly relationship between teachers and students. Such a strategy is highly effective in promoting students sense of academic self-efficacy and perceived campus belonging, which greatly increases their involvement in learning. At the same time, it also enables college students to have a clear understanding of the synergistic effects between external environmental support, personality characteristics, and learning resilience. It motivates them to put more focus on building learning confidence and actively work on improving their ability to overcome failures. Through the development of learning resilience as a way of coping with academic problems and adversity personally, they are able to develop self-esteem in a positive learning environment and social connections. The procedure helps them to know themselves, acknowledge their worth and go beyond their constraints, and eventually reach higher scores in academics.

Certainly, some findings require cautious interpretation. In the present sample, the direct path from learning environment to academic self-efficacy did not reach the conventional 0.05 level of significance (*β* = 0.123, *p* = 0.071), and the direct path from learning environment to learning resilience was also non-significant (*β* = 0.026, *p* = 0.550). Likewise, the indirect path through academic self-efficacy was positive but not significant at the 0.05 level (*β* = 0.053, *p* = 0.079). However, learning environment was significantly associated with perceived campus belonging, and it showed a significant indirect association with learning resilience through perceived campus belonging (*β* = 0.094, *p* < 0.01). Therefore, the present findings do not support a strong direct effect of learning environment on learning resilience, but neither do they justify the conclusion that learning environment is entirely unrelated to learning resilience. Instead, the results suggest that its association with learning resilience may operate more clearly through perceived campus belonging than through academic self-efficacy in this sample.

## Research significance

### Theoretical contributions

The present study will explore the mechanisms of generating individual characteristics (academic self-efficacy, perceived campus belonging) of college students in various external learning environments and their impact on academic resilience via empirical strategies. We are now able to understand in greater detail the processes of formation and contextual bases of learning resilience in college students, due to the development of the structural model of the relationship between an external context, individual traits, and academic resilience. The model provides a theory-informed framework for understanding how external contextual perceptions, academic self-efficacy, perceived campus belonging, and learning resilience are statistically related in the present sample. In particular, our study suggests that students’ perceptions of academic and social support are associated with academic self-efficacy and perceived campus belonging, which in turn are associated with learning resilience, although the strength and significance of these paths differ across specific contextual dimensions. This result broadens the contextual explanatory ability of the learning resilience theory and enriches the comprehension of the mechanisms of resilience formation. At the same time, incorporating the triadic interaction theory, we have successfully developed a comprehensive model of the relationship between external environment, personality traits and learning resilience. Taken together, these findings refine the contextual explanation of learning resilience and clarify the mediating roles of academic self-efficacy and perceived campus belonging within the external context–individual characteristics–learning resilience framework. By integrating social cognitive theory and triadic interaction theory into a single structural model, this study provides a more differentiated account of how distal contextual perceptions are associated with learning resilience through more proximal psychological mechanisms.

### Practical contributions

The practical contribution of this study lies not in demonstrating strong direct leverage of external context on learning resilience, but in identifying several context-related factors that are indirectly associated with learning resilience through academic self-efficacy and perceived campus belonging. Therefore, the findings offer cautious rather than strong recommendations for educational management. Specifically, the findings suggest that universities may be more effective in supporting learning resilience when they focus on strengthening academic self-efficacy and perceived campus belonging through a positive learning climate and supportive social relationships, rather than assuming that the general learning environment alone will directly enhance learning resilience. In practice, this implies that teachers and administrators may consider providing constructive feedback, creating opportunities for peer collaboration, encouraging classroom participation, and fostering inclusive teacher–student and peer relationships. These practices may help students maintain confidence in academic tasks, regulate negative emotions, and sustain persistence when facing academic setbacks. At the same time, these implications should be interpreted cautiously, because the direct effects of learning environment and learning climate on learning resilience were not statistically significant in the present sample.

### Limitations

Even though our study produced significant findings, the sample was restricted to particular areas and kinds of institutions so these findings can be not entirely representative and explanatory of the situations of university students in various areas. Since the current study has a cross-sectional design, we could not adequately reflect the dynamism of individual attributes and academic resilience. More importantly, because all variables were measured at one point in time, the SEM results cannot be interpreted as evidence of causal effects, but only as theory-consistent statistical associations among the studied constructs. It means that our findings cannot cover all the real experiences of students with those changing processes. Even though we try to measure the variables of the study (learning climate, social relationships, etc.) correctly, there will be some measurement errors. This difference is possibly explained by flaws in questionnaire construction, differences in the understanding of respondents, or any other possible factors that could have an effect on academic self-efficacy and learning resilience in a multifaceted learning environment.

### Research outlook

Future empirical studies in the field will increase its sample size in order to make the results more universal and representative, including a wider geographic region or a more varied range of higher education institutions. This allows us to learn more about the personal features and learning resilience of university students in various situations, thus, discover more universal tendencies of behavior. In addition, the longitudinal tracking research designs are needed to obtain a better insight into the dynamic interaction between the personal attributes and learning resilience. By conducting long-term data gathering, we can better illustrate the actual experiences of students and developmental paths during their learning process, and consequently make more specific and timely suggestions to the educational practice. Moreover, learning resilience intersects with intrinsic motivation, which finally shapes academic engagement and academic achievement.

The relationship system between external context-individual traits-learning resilience can be expanded to create a cyclical model of learning engagement and academic achievement in future research. On the issue of measurement tools, future studies should be constantly optimized and improved in terms of measuring variables to minimize errors and enhance research accuracy. It involves other things like coming up with more reliable and valid questionnaire items and combining various forms of data gathering tools such as interviews and observations so as to attain accurate and reliable research results. Lastly, it is important to investigate other possible factors that could impact individual traits and learning resilience in an attempt to develop a more elaborate theoretical framework. Not only does this enrich our knowledge of college-going students learning behavior, but also offers a sounder scientific foundation to the formulation and implementation of educational policies so as to advance and sustain education development.

## Data Availability

The original contributions presented in the study are included in the article, further inquiries can be directed to the corresponding author.
